# A longitudinal, qualitative exploration of women’s sexual recovery following surgical repair of pelvic organ prolapse

**DOI:** 10.1177/17455057261437391

**Published:** 2026-04-20

**Authors:** Kaylee Ramage, Erin A. Brennand, Lauren M. Walker

**Affiliations:** 1 Department of Public Health, College of Education, Health, and Human Sciences, University of Tennessee Knoxville, TN, USA; 2 School of Public Health, College of Health & Human Services, San Diego State University, CA, USA; 3 Department of Obstetrics & Gynaecology, Cumming School of Medicine, University of Calgary, AB, Canada; 4 Department of Community Health Sciences, Cumming School of Medicine, University of Calgary, AB, Canada; 5 Department of Oncology, Cumming School of Medicine, University of Calgary, AB, Canada; 6 Department of Psychology, Faculty of Arts, University of Calgary, AB, Canada

**Keywords:** pelvic organ prolapse, sexuality, surgery, qualitative analysis

## Abstract

**Background:**

Pelvic organ prolapse (POP) occurs when one or more female pelvic structures descend from their anatomic position into or through the vagina. Previous quantitative research has shown that POP may adversely affect sexual activity, with mixed results regarding the impact of surgical repair of POP on women’s sexual activity.

**Objectives:**

Our study addresses the need for longitudinal, qualitative research centering women’s lived experiences of POP, examining women’s sexual activity before and after POP surgery.

**Design:**

Qualitative.

**Methods:**

We conducted remote, longitudinal, qualitative interviews with women experiencing POP across three time points: prior to surgery, 3 months post-surgery, and 6 months post-surgery.

**Results:**

Three major themes emerged elucidating women’s return to sexual activity after surgical repair of POP, experiences with sexual activity prior to and post-surgery, and sexual outcomes and rewards experienced prior to and post-surgery. Women’s expectations for sexual activity post-surgery were not always achieved, and recovery time was often slower than anticipated; however, most women reported improved sexual activity by 6 months post-surgery.

**Conclusion:**

Our study highlights the complexity of women’s sexual experiences before and after POP surgery. Clinical counseling around the impact of surgical repair of POP on sexual activity should include expectation management, including the timeline for recovery and which aspects of sexuality may improve.

## Introduction

Pelvic organ prolapse (POP) occurs when one or more female pelvic structures (i.e., bladder, uterus, small bowel, and rectum) descend from their anatomic position into or through the vagina.^1^ For many, POP is associated with negative impacts on women’s daily lives, including emotional distress, poor body image, urinary or fecal incontinence, seeing a bulge or protrusion from the vagina, and/or pelvic pressure.^2–6^ In addition to these impacts, POP can be associated with significant sexual dysfunction.^7,8^ Those living with POP may experience symptoms related to sexual activity, including physical discomfort or pain associated with sexual intercourse (dyspareunia), urinary incontinence during sexual activity, difficulty with orgasm, decreased sexual satisfaction, and increased anxiety associated with intercourse, or avoidance of intercourse and/or other sexual activities.^7,9^

Although POP affects approximately half of childbearing women in their lifetime, little is known about its impacts on women’s sexuality, sexual function, and sexual relationships. Most existing literature on sexuality for women with POP focuses on exclusively on the measurement of sexual function and anatomy, rather than on quality-of-life-related outcomes^10^ or other important indicators of sexual well-being. Furthermore, quantitative assessments often fail to adequately capture the nature of women’s sexual difficulties.^11^ The gold standard for sexuality research within women’s experience of POP is a single, condition-specific, validated measure of sexual function for use with POP patients, the Pelvic Organ Prolapse/Urinary Incontinence Sexual Function Questionnaire (PISQ).^12^ This tool exists in both long and short forms and has been used in numerous studies examining the association between POP and women’s sexuality.

Quantitative examinations of sexuality and sexual function across POP populations demonstrate mixed results, highlighting the need for examination of why reported differences may occur, and whether patient characteristics or experiences may influence sexual outcomes. For example, Lukacz et al. demonstrated that PISQ scores improved over time after surgery, but that frequency of sexual activity did not change significantly over time.^13^ Conversely, a retrospective study of women undergoing POP surgery (*n* = 151) documented increases in sexual activity and decreases in dyspareunia post-surgery.^14^ In another study (*n* = 1267), women with genital prolapse had lower PISQ-12 scores than women without POP, indicating worse sexual function. In this same study, the authors identified additional factors negatively impacting sexuality that were not assessed with the PISQ-12, including urinary incontinence during sexual activity, fear of incontinence, and avoidance of intercourse due to prolapse. These results conflicted with studies documenting no differences in orgasm or changes in sexual satisfaction among women with POP.^15^ Other studies have noted differences in sexual function related to POP severity such as lower sexual desire, sexual arousal, and orgasm during intercourse, in women with stage 2 or greater POP compared to women with stage 0 or 1 POP.^16^

Sexuality is an important part of women’s lives, which can be affected or even disrupted by the experience of POP. Measurement of the impact of POP on sexuality through standardized, validated questionnaires assessing sexual function, such as the PISQ-IR is important; however, these tools are limited in scope of assessment and, therefore, provide little understanding as to the process of *how* POP and POP surgery may impact women’s sexuality. Furthermore, quantitative measures also overlook the voices of women and fail to recognize the nuanced ways that POP and treatment can affect sexual activity and sexuality. Despite this gap in the literature, qualitative research examining the impact of POP on women’s sexuality is sparse. One recent mixed-methods study by Shah and colleagues (*n* = 16)^17^ examined the experiences of sexuality and sexual activity in women with POP, finding that POP affected desire, arousal, sexual satisfaction, and women’s motivation to engage in sexual activity. However, to our knowledge, no qualitative research has examined women’s sexuality both before and after surgery to repair POP.

Women seeking treatment for POP, and clinicians providing care for these women, need information regarding how sexuality changes after surgery and how surgical treatment for POP may affect their sexuality, requiring both longitudinal assessment and methodological approaches. Given the limitations of using only quantitative measurement of sexual function,^18^ qualitative assessment provides the opportunity for more nuanced understanding of the complexities of sexual impacts related to prolapse both pre- and post-surgery. Anecdotally, women have indicated that they are seeking a richer understanding of sexuality post-surgical repair for POP, but that the shame and stigma related to POP stifles conversations women might have with others in order to gain this information. To address this gap in the literature and to provide evidence for women looking for more detailed understandings of sexuality following surgical repair for POP, the current study aims to center women’s voices in a longitudinal qualitative examination. The primary focus of this study is to understand: (1) women’s experiences of sexuality while living with POP, (2) their expectations associated with anticipated surgery, and (3) their experiences of sexuality during the 6 months following POP surgery.

## Materials and methods

This study adheres to the Standards for Reporting Qualitative Research.^19^

### Sample eligibility and recruitment

Women who were scheduled for surgery to repair POP were recruited from the Calgary Pelvic Floor Clinic at Foothills Medical Centre (Calgary, Alberta, Canada) between February and August 2021. Potential interview participants were invited from those who had already agreed to take part in a larger multi-pronged, mixed-methods study evaluating surgical POP treatments.^20^ Eligibility criteria included having: a prolapse of grade 2 or greater (as defined by the Pelvic Organ Prolapse Quantification (POP-Q) measure)^21^ (see Table 1), elected surgical management of their POP, no history of hysterectomy, and no desire for further pregnancy. Participants also needed to be English proficient, 18 years or older, and have a history of sexual activity prior to their surgery. Women who did not follow through with surgery (e.g., did not meet the clinical criteria, canceled their surgery, and opted for non-surgical management of their POP) were excluded from analysis. Ethics approval for this project was received from the University of Calgary’s Conjoint Health Research Ethics Board (REB19-2134). All participants provided written informed consent for their participation in this study.

**Table 1.** Pelvic organ prolapse quantification.

Stage	Description
Stage 0	No prolapse demonstrated
Stage 1	The most distal portion of the prolapse is >1 cm above the level of the hymen
Stage 2	The most distal portion of the prolapse is 1 cm or less proximal or distal to the hymenal plane
Stage 3	The most distal portion of the prolapse protrudes >1 cm below the hymen but protrudes no farther than 2 cm less than the total vaginal length
Stage 4	Vaginal eversion is essentially complete

### Study design

To measure changes in women’s experiences over time, we used a longitudinal qualitative design, which specifically incorporates time and change into the analysis and allowed us to look at the possible impact of an event (i.e., surgery) on the phenomenon of interest (i.e., sexuality with POP). For the purposes of our study, qualitative longitudinal research allowed for repeated interviews with the same individuals over time^22^ and, we aimed to make “***change*** a central focus of analytical attention” (p. 185).^23^

Interviews were conducted prior to women’s surgery (“baseline” interview), at 3 months post-surgery (“3-month”) and at 6 months post-surgery (“6-month”). As one of the goals of this research was to understand the impact of surgery on women’s sexuality with POP, it was important to capture women’s experience of sexuality prior to their surgery in order to understand how surgery may have changed their experiences. The 3-month interview was scheduled to occur after the conventionally prescribed healing period for surgical repair of POP of approximately 6–8 weeks (when follow-up exams typically clear women for return to physical and sexual activity). It was assumed that participants would have opportunity to return to sexual activity during the gap between the post-surgical follow-up visit (occurring at 6–8 weeks post-surgery) and the 3-month interview. The 6-month interview was meant to capture women’s experiences post-surgery, wherein women may have had the opportunity to return to sexual activity, as physical and sexual recovery following surgery was thought to be more likely.

Interviews were conducted via telephone or Zoom, depending on participant preference, and took place after the participant elected for surgical management of their POP and after the participant’s surgery. Both the interview and the analysis were guided by the research question, focusing on how women’s experience of sexuality may be impacted by both POP and surgical repair. Interview questions aimed to facilitate in-depth dialogue for the participant regarding their experiences of POP, sexuality, surgery, and recovery as well as intersections thereof. Interviews were audio-recorded with participant permission (one woman opted for notes to be taken instead of audio-recording). All participants were assigned a pseudonym. Baseline data collection was stopped when saturation was determined to be reached, where new data are redundant of data already collected.^24,25^

### Data analysis

Our analysis followed a Framework Approach,^26,27^ a type of thematic analysis often used in interdisciplinary heath research,^26^ to allow for within- and across-case analysis over time. The Framework Approach uses five steps. Interview recordings are first transcribed verbatim; then, the research team familiarizes themselves with the interviews by reading/listening and repeating as necessary. Interviews are then coded using a line-by-line methodology; next, an analytical framework is developed, where codes are grouped together into categories, and last, the analytical framework is applied to each transcript with the data charted into the framework matrix by participant and across time (i.e., for each participant, the codes for the baseline, 3-month, and 6-month interviews were charted). Finally, data were interpreted by the research team, considering experiences over time, both within and between participants, and in the context of the broader literature.

### Reflexivity statement

Researcher’s previous experience with the research topic may have influenced the study design, data collection, analysis, and interpretation; however, efforts were made to stay consistent with the specific words and reports of the research participants. As none of the research team had personally experienced surgery for POP, all held positions as “*informed outsiders*.” To reduce potential for personal beliefs and perceptions influencing the research, the research team critically reviewed the research findings and interpretations and triangulated their perspectives.

## Results

Longitudinal interviews were conducted with 18 women who underwent surgery for POP repair (either hysterectomy-based or uterine-preserving surgery). An additional four participants were excluded from the analysis – two who completed the baseline interview and surgery but were lost-to-follow-up, and two who completed the baseline interview but did not have a surgery. Sociodemographic characteristics of the participants are available in Table 2. All participants identified as white, heterosexual women. Most were married or in a common-law relationship. Half had completed the menopause transition. At baseline, participants’ average POP-Q score was 2.2.

**Table 2.** Participant sociodemographic characteristics at their baseline interview (pre-surgery) (*n* = 18).

Sociodemographic characteristic	Mean (range) or *N* (proportion)
Age	51.4 years (31–75 years)
Race/Ethnicity	
White	18 (100.0%)
Gender	
Woman	18 (100.0%)
Sexual orientation	
Heterosexual	18 (100.0%)
Marital status	
Married or in a common-law relationship	16 (88.9%)
Widowed	1 (5.6%)
Separated	1 (5.6%)
Educational level	
Completed high school	2 (11.8%)
Completed some post-secondary	4 (23.5%)
Completed post-secondary	8 (47.1%)
Completed graduated or professional degree	3 (17.6%)
Annual household income	
<$50,000	2 (11.8%)
$50,000–$80,000	3 (17.6%)
$80,000–$100,000	4 (23.5%)
$100,000–$200,000	5 (29.4%)
>$200,000	3 (17.6%)
Menopause status	
Completed menopause	9 (50.0%)
Have not completed menopause	9 (50.0%)
POP-Q score at baseline	2.2 (2–3)
Number of children	2.1 (0–4)

POP-Q: Pelvic Organ Prolapse Quantification.

Overall, three major themes emerged: (1) women’s return to sexual activity post-surgery; (2) experiences with sexual activity prior to and post-surgery; and (3) experiences related to sexual outcomes and rewards prior to and post-surgery (see Table 3). Each of these themes had several sub-themes, as discussed below. Throughout, where relevant, we reference women’s baseline experiences (pre-surgery) to inform interpretation of their post-surgery experiences.

**Table 3.** Major qualitative themes and sub-themes.

Women’s return to sexual activity
Timeline for return to sexual activity Anxiety Pain More time needed for healing Sexual initiation Willingness and capacity for sexual activity Expectations for post-surgery sexual activity
Women’s experiences with sexual activity
Mental comfort and discomfort with sexual activity Sexual desire and arousal Types of sexual activities Physical sensations associated with sexual activity
Sexual satisfaction & rewards after surgical repair of prolapse
Improved orgasm quality Rewards gained from sexual activity

### Women’s return to sexual activity after surgery

Several themes related to women’s return to sexual activity after POP surgery emerged, including the expected and actual timeline for return to sexual activity, sexual initiation and receptivity, and how returns to sexual activity met or did not meet expectations.

#### Timeline for return to sexual activity

Women were largely cleared by their physician to return to sexual activity at the clinically-recommended 6–8 weeks post-surgery. While the majority of women indicated at baseline that they expected to follow this timeline, many women reported delaying their return to sexual activity. Several reasons for this delay emerged, including anxiety, pain, and needing more time for healing.

Many women experienced anxiety and nervousness about potential pain and discomfort, and worried that sexual activity might negatively affect the healing process or “*wreck the surgery*.” Josephine noted, “*I was nervous, not knowing what to expect*,” and after a first attempt at intercourse, she decided to allow herself more time to recover before resuming again. She had clearance from her surgeon, no bleeding, was reportedly “*feeling good*” and like her sutures had healed, however, her first experiences post-surgery was accompanied by tenderness and some bleeding. She then waited an additional few weeks before trying again, saying that “*[we] just spaced it out to make sure that we’re not doing something we shouldn’t be doing yet*” and that “*I just better be careful*.” She similarly reported: “*I want to make sure I don’t damage that area with vaginal penetration*.”

At the 3-month interview, several women noted they had experienced pain and discomfort with their initial return to sexual activity in previous weeks, including muscle tension or tenderness. By 3 months, reports of pain often dissipated with many women describing a return to “normal” sexual activity. However, this was not the case for all. For example, at her 3-month follow-up interview, Amy noted that “*sex is nowhere near what it used to be because I’m still afraid*” but by 4–5 months post-surgery, sex was “*great*” and “*like before I had kids*.”

Finally, at the 3-month interview, several women were not completely healed from surgery and needed more time to recover. For example, Hannah attempted to return to sexual activity at 8 weeks but was uncomfortable, so decided to wait several more weeks before trying again. She noted that there was also “*a bit of paranoia too, because you’re not sure how well the stitches inside healed [and] I don’t want anything to be snagged*.” By 3 months post-surgery, she reported “*we’re back to normal [and]. . . it’s definitely an improvement*” but still shared that she was worried about a slow-dissolving stitch which was causing pain and was concerned it might be a permanent issue. By 6 months however, this issue resolved and Hannah noted “*I think maybe it just needed more time to heal*.” By the 6 month interview, more women were sexually active than at the 3 month interview.

#### Sexual initiation

At their baseline, many women had reported that physical and mental impacts of POP (e.g., pain and lack of confidence) reduced initiation of partnered sexual activity. Catherine said that prolapse made her “*somewhat more hesitant to be sexual spontaneously with [her] partner*.” Claire noted that she never initiated sexual activity because of pain and decreased sexual desire, asking “*why would I want to be spontaneous – would you choose to punch yourself in the face?*” Sophie shared that she felt guilty for not initiating sex and for her lack of desire for sex, stating that “*it’s not because I don’t love my husband or I’m not attracted to him, it’s more just because I’m not comfortable with myself*.” Post-surgery, many women reported slow and “*cautious*” returns to sexual activity and sexual initiation in accordance with the healing process. For example, at her 3-month interview, Audrey noted that she was still not able to be sexual because the recovery from surgery was so painful, but “*once I got the ‘okay’ [from the surgeon], we were going forward cautiously*.” Most women noted that, once they had recovered physically from the surgery, their initiation of sexual activity increased. Sarah noted that she “*wanted to have sex more*” and Hannah noted that her willingness to initiate sex had “*definitely improved*,” with an increase in “*freedom*” without the worries of painful sex. Factors beyond the surgery also had an impact on women’s sexual initiation, including shifts in partner dynamics, changing living situations, and having young children.

#### Willingness and capacity for sexual activity

At baseline, most women reported increased hesitation and low sexual receptiveness to invitations from their partner, often attributed to physical and mental aspects of POP. Hannah noted that POP had made it “*a bit more tricky*” because if “*I don’t feel as confident, then I might be less inclined to want to participate [in sexual activity]*.” Similarly, Sophie stated that she was definitely more “*resistant or [would] just try to avoid it*” because it was not enjoyable. Amy reported that sex was “*not happening because I don’t want it to happen because it’s no fun for me. . . it’s just more of an avoidance at this point*.” She also reflected that her husband was not initiating as much as he normally would because “*no one likes getting rejected*.” Olivia reported that her husband had “*just stopped asking for it or wanting it or whatever*” and that sexual activity had stopped. Indeed, some women discussed a total lack of receptiveness and avoidance of sexual activity, whether by themselves or with their husbands. In contrast, some women were still receptive to partner’s initiations. For example, Sarah shared that while she did not feel sexual and did not “*care to have sex*,” that [her partner] was “*still attracted and willing, [therefore] I’m fine. . . and I’m willing*.” By 3 months post-surgery, most women reported that their receptiveness to sexual invitations from a partner had improved. However, for some women, the process took longer. For example, at 3 months post-surgery, due to the tenderness related to her recovery, Amy was still hesitant and reported that her partner “*might get turned down a little more often*”; however, by 6 months, she stated, “*I actually want to* [engage in sexual activity].” However, even at 6 months, there remained a portion of participants who were still not very receptive to sexual invitations. At her 6-month follow-up interview, Claire reported that receptivity to her partner’s initiations had not really changed, saying that “*I’m a little blasé about sex right now*.” Kelsey shared that, despite experiencing a return of sexual desire, her interest in sexual activity had not returned, as she was still experiencing pain and pelvic floor muscle tension even 6 months post-surgery.

#### Expectations for post-surgery sexual activity

Beyond expectations related to the timing for their comfortable return to sexual activity post-surgery, other expectations related to sexual activity emerged. At their baseline interview, several women acknowledged that their prolapse was one of many factors that affected their sexuality and sexual behavior. One woman mentioned that “*[POP] is not the only factor. . . it’s hard to segregate exactly how much of that is prolapse and how much of it is just the joys of motherhood*.” Women mentioned other influencing factors such as having young children, being exhausted, conflicting work schedules, living situations, relationship issues, and their partner’s erectile difficulties. Even with the knowledge that sexuality and sexual activity are influenced by multiple factors, several women mentioned feeling guilty about the quality and frequency of their pre-surgery sexual activity and largely related it to their prolapse. For example, Audrey stated that she felt like her infrequent sexual activity failed to meet her “*wifely duties*” and she felt she was “*letting [her husband] down*.” Hannah worried that sex was not as good for her partner with the presence of the prolapse. Claire noted that her lack of pleasure during sexual activity affected her partner’s ego and emotional response to sex. Jane attributed her partner’s difficulty with reaching orgasm to her vaginal laxity, stating that “*I’ve always figured that [his difficulty having an orgasm] was because of the prolapse, that I’m a lot looser and it’s not as sexy and desirable and he’s not achieving [orgasm]*.” The subtext under a lot of these comments was the hope that their sexual relationship would improve following surgical repair of POP. Whereas before surgery, women had often placed the blame for the reduced quality of their sexual activity on their prolapse, many concerns remained even after their surgery and recovery. For Jane, even with her “*new and improved vagina*,” her husband still had difficulty achieving an erection which negatively impacted both parties’ satisfaction with their sexual relationship. At baseline, she expected that her sexual desire would improve following surgery, but at 6 months, she noted, “*it’s very difficult to get aroused*” and mused that it might be related to decreased frequency of sexual activity and to her husband’s erectile dysfunction. Claire mentioned that, despite increased sexual desire and satisfaction with sexual activity, the frequency of her and her partner’s sexual activity had not increased and related this lack of increase to several factors including: surgical recovery, their current living situation, having small children, and her feeling somewhat indifferent about sex. Other women discussed the effects of medication such as anti-depressants on arousal and ability to orgasm, with one woman stating that her orgasm had been “*zapped*” by her medication.

### Women’s experiences with sexual activity

In the following section, we discuss women’s experiences with sexual activity after surgery, including mental comfort and discomfort, sexual desire and arousal, capacity for types of sexual activity, and physical sensations associated sexual activity post-surgery.

#### Mental comfort and discomfort with sexual activity

Prior to surgery, many women noted mental barriers to sexual activity, associated with the experience of prolapse, including body image concerns, perceived undesirability, lack of confidence, self-consciousness, and embarrassment associated to the POP. Amy explained that the mental aspect of prolapse was stronger than the physical, saying that “*with my prolapse, I don’t feel sexy anymore, like I don’t want my husband touching me, I don’t want him going near my vagina*.” Debbie’s symptoms of prolapse led to an avoidance of sexual activity, “*once [she] noticed the bulge*,” she felt “*very self-conscious when it comes to any kind of intimacy*.” Sarah too noted that prolapse “*made me feel less attractive and desirable. . . I don’t feel sexual, I don’t feel desirable, I’m just embarrassed about [the prolapse]*.” Post-surgery, however, women’s body image generally improved and, without the physical aspects of POP, women were less embarrassed about their genitals and body during sexual activity. Christine noted that “*sex was a lot better because I wasn’t actually worried about the prolapse*.” Sarah noted that post-surgery, “*things look much nicer down there. . . I feel better about myself*.”

#### Sexual desire and arousal

Pre-surgery, approximately half of the women reported that their POP had not resulted in changes in sexual desire, while the other half noted that sexual desire decreased with the onset and worsening of their POP. For Olivia and her partner, prolapse had “*flattened everything*,” leading to decreased sexual desire and a subsequent loss of intimacy. Similarly, Jane mentioned that because “*things aren’t as pleasurable. . . it can affect spontaneous desire a lot*.” Post-surgery, most of the women who had noted decreased sexual desire pre-surgery reported that sexual desire had improved. For example, Mary noted that post-surgery she was more “*ready*” to have sex. For many women, arousal was reportedly lower pre-surgery, but improved post-surgery. Christine noted that it was “*easier to become aroused*” and other participants specifically noted improved pleasure, which helped to facilitate more arousal. While Patricia no longer engaged in penetrative intercourse with her partner, she noted increased sexual “*urges*” post-surgery that were not present with the POP. Despite reports of improved subjective arousal, many women also reported vaginal dryness and increased need to use sexual lubricants post-surgery.

#### Types of sexual activities

Pre-surgery, about one-third of the women did not report negative impacts of POP on sexual activity, but most women (*n* = 11) stated that prolapse had reduced the variety of activities they were willing and able to engage in. In particular, this included changes in sexual positions to accommodate the prolapse (i.e., a bulge) and avoidance of oral sex or other types of vulval or vaginal touch. Women often reported avoiding oral sex due to embarrassment that their partner might see and be disturbed by a noticeable or visible bulge. After surgery, most women who had returned to sexual activity noted that they were able to return to the same types of activities in which they had engaged prior to the prolapse. Some of the women noted that the return to “*normal*” sexual activity post-surgery took more time than initially expected and resulted in reduced variety of sexual activities. Audrey reported that, at 3 months post-surgery, she was still healing and was “*exploring and trying new positions again and seeing what works and what is still a little off*,” but by 6 months post-surgery, she was back to almost all pre-prolapse activities. Similarly, Amy noted at 3 months post-surgery she was “*sticking to the basics*,” but by 6 months post-surgery, she was back to normal sexual activity with her partner “*anytime, anywhere*.” Several women noted that post-surgery, their sexual activity returned to include oral sex and other types of vulval or vaginal touch. Claire reported that she was able to be more adventurous with sex and found it to be more enjoyable. The post-surgery change in the sensation of sexual activity for Hannah had impacted the types of sexual activities she engaged in; in her interviews, she indicated that she had been self-conscious about the prolapse pre-surgery and that post-surgery, she could “*feel more*” and she was “*more inclined to try more things*,” instead of sticking to the same positions.

#### Physical sensations associated with sexual activity

Prior to surgery, some women reported that prolapse affected the physical sensations associated with sexual activity. Women noted sensations associated with a “bulge,” vaginal laxity or “looseness,” lack of sensitivity or sensation, and pain. In particular, women noted differences in friction during penetrative intercourse, vaginal dryness, and “different” sensations during sexual activity. For example, prior to surgery, limited sensation was attributed to vaginal laxity and reduced or “less” friction and conversely, too much friction associated with certain positions resulted in pain. Post-surgery, many women noted increased satisfaction with the friction and contract during intercourse. Josephine noted more “*vaginal sensitivity*” and Steph noted that the friction was “*back to normal*.” Many women also noted some tenderness or discomfort associated with healing from the surgery. For example, Claire commented that sex was “*painful [because] things were new and in different places*,” but that this seemed to be improving with increased sexual frequency. Sophie noted that sex was “*fine*,” but she could still feel her stitches and felt pressure on her scar tissue. By 6 months, most of the women described resolution of pain and discomfort. However, Kelsey still reported severe pain and pelvic floor tightness and was unable to engage in sexual activity without pain. Vaginal dryness and lack of lubrication post-surgery were also common.

### Sexual outcomes and rewards after surgical repair of prolapse

Women often reported improved orgasms and finding sexual experiences to be more rewarding following surgery.

#### Improved orgasm quality

Most women noted an “*improvement in orgasm*” compared to pre-surgery, potentially related to increased capacity for return to oral sex and other types of genital touch, or to increased pleasurable sensations associated with sexual activity. Before their surgery, most women had noted that their orgasm had been negatively impacted, and was often less frequent or intense, or more difficult to achieve through the same activities. Some women attributed this to the physical changes or pain during sex that had emerged with the POP. Claire reported that “*pain tends to stop you in your tracks*,” so orgasm does not happen as often. Sophie related that she could not “*remember the last time I had an orgasm, probably because there’s all this stuff in the way*.” Kelsey noted that prior to prolapse, she used to have very intense orgasms but with POP it was not the same, stating that “[now] *it’s just nice, that’s about it. Before [POP], it was like, whoa!*” For women who had returned to sexual activity post-surgery, most women reported that their orgasm had returned to pre-prolapse levels or was slightly better. Amy noted that her orgasm had improved and was both stronger in duration and intensity. Some women attributed their post-surgery improvements in orgasm, more to the absence of negative mental impacts of prolapse and others to increased capacities for more effective types of stimulation (e.g., oral sex). In contrast, some women reported difficulties with their orgasm post-surgery. Claire noted that her orgasms were different post-surgery, with orgasm occurring only with clitoral stimulation, not through penetration. Jane mentioned that she found it very difficult to achieve orgasm at all, post-surgery.

#### Rewards gained from sexual activity

Women noted reductions in the rewards gained from sexual activity related to their POP with disruptions of the emotional and physical connections with their partners. Prior to surgery, many women discussed decreases in the physical rewards of sex with prolapse affecting their sexual initiation and receptiveness. They described pain, reduced pleasure, challenges with orgasm, embarrassment, and self-consciousness, often hindering their capacity for rewards and enjoyment. For many women, upon their return to sexual activity post-surgery, physical and emotional rewards from sexual activity increased, linked to increased sexual arousal and desire, which, in turn, facilitated more enjoyable sexual experiences in which they were then more motivated to take part. With the absence of pain and discomfort associated with prolapse, many women noted increased physical pleasure. They also noticed being more comfortable with the kinds of activities they were able to engage in, and less pre-occupied with worries about their partner noticing the POP. They reported increased confidence to engage in activities and being more able to “*fully engage*” and “*pay attention*” to sexual activity compared to pre-surgery.

## Discussion

To our knowledge, this longitudinal, qualitative study is one of the first to analyze women’s lived experiences related to sexuality after surgical repair and recovery for POP in relation to their pre-surgery experiences and to center women’s voices using a qualitative approach. Women’s experiences with POP were diverse, affected by POP symptoms and severity, partners, and surgical experiences, but similarities were seen across these experiences. Prior to their surgery, many women reported pain and discomfort with sexual activity, limits to their physical pleasure, and decreased sexual confidence and negative body image due to the bodily changes from POP. Over time, with the recovery from POP repair, many women’s sexuality and sexual activity realigned with their experiences before POP; however, the timeline for the return to “normal” sexual activity was often longer than anticipated. Women also typically expected that much of their reported difficulties with sex pre-surgery would be improved with the surgery; however, most participants shared that even though their POP had been repaired and was, therefore, less of a cause of their concerns, many sexual concerns still remained.

Women’s sexuality is extremely nuanced and the experience of POP can impact women’s sex life, both physically and mentally. Our results regarding women’s experiences of sexuality with POP were similar to the findings of a recent mixed-methods study that focused on women beginning pelvic floor physical therapy for prolapse,^17^ which noted that women with POP had reduced sexual initiation and receptiveness, reduced arousal and sexual desire, and that POP had a negative impact on genital satisfaction. Similar to other studies of women’s chronic illness or injury and sexuality,^28–30^ POP impinged upon women’s sense of sexuality and femininity, affecting beliefs about their adequacy as women and partners. A recent qualitative examination of sexuality and body image in women following amputation found both primary (e.g., loss of body part) and secondary (e.g., weight gain, decrease in physical activity, and sense of self) impacts of amputation, which mirrors the POP of prolapse on women’s sexuality.^31^ Similarly, an analysis of women’s experiences of anal incontinence following obstetric anal sphincter injury found that women felt significant loss of identity and negative psychological impacts due to their injury and were trying to retrieve a sense of normality in their lives.^32^ In the current study, women had chosen surgery as a pathway to achieving that normalcy. Like these other injuries, POP had both an immediate and broad effect on women’s sexuality for which they desired intervention.

Similar to quantitative studies examining sexuality pre- and post-surgical repair for POP,^18,33,34^ the impact of surgery was generally positive, with most women reporting resolution of their symptoms of POP, increases in sexual confidence and desire, and less pain and discomfort during sexual activity. However, expectations of the impact of surgery on sexuality and sexual activity, where surgery would “fix” all of their issues and return their sex lives to “normal,” were not always met, similar to other research on elective surgeries.^35^ Women’s experience of POP left them feeling broken and inadequate, and their expectations for post-surgery that these issues would be resolved. Our longitudinal analysis also examined how women’s experience with POP affected their perceptions of sexuality post-surgery, with fear of pain and of damaging the surgical healing process preventing them from re-engaging in sex. Post-surgery, women typically needed more time than the recommended 6–8 weeks to recover physically and feel mentally prepared to engage in sex. Additionally, their expectations that their sex lives would recover following POP correction were often not fully realized, as, in the larger context of the women’s lives, POP appeared to often be only one of several factors negatively impacting sexuality. Overall, however, the majority of the sample did report improvements in sexual function.

Through our study results, it was revealed that women undergoing surgical repair of prolapse may have unrealistic expectations regarding the extent to which surgery may solve their sexual difficulties, given our finding that many may attribute all of their sexual issues pre-surgery on prolapse and disregard other factors. There is a need for pre-surgical discussion to foster more appropriate expectations regarding sexual improvements after prolapse repair. Clinical counseling should recognize that the impacts of POP on women’s sexuality go beyond the physical (i.e., bulge, dyspareunia, incontinence, etc.), and that the mental and emotional impact can be primary and may remain following surgical repair. Furthermore, given the surprises women reported during the recovery from surgery (whether related to sex or otherwise), the process of surgical recovery may also present additional limitations to sexuality. Advice regarding capacity to resume sexual activity approximately 6–8 weeks post-surgery did not appear to fit for many women because of continuing pain, slow-dissolving stitches, or pelvic floor muscle tension. As well, even women who were physically healed at 6–8 weeks post-surgery reported feeling tentative or nervous to return to sexual activity. This aligns with the postpartum literature on returning to sexual activity after childbirth, where women’s experiences of sexuality are often disrupted by the trauma of childbirth and realities of child-rearing and postpartum recovery^36,37^ and only approximately one-third of women return to sexual activity at 6–8 weeks postpartum.^38^

In addition to adequately preparing patients to manage expectations about sexual changes and recovery, clinicians would do well to provide patients with information about resources that present an integrated and biopsychosocial model to sexual rehabilitation. Indeed, a recent comprehensive review describes sexual rehabilitation strategies following abdominal or pelvic surgery, including hysterectomy and POP repair. Interdisciplinary sexual rehabilitation should emphasize pelvic floor muscle training and manual therapies, psychological, relational, and sexual counseling and may also involve medication or supplementation.^39^

Given the complexities evident in our analysis and other studies, there is a clear need for qualitative research in women’s health and, more specifically, urogynecological research. Other research on sexuality and sexual rehabilitation for patients undergoing prolapse repair exists,^39^ but the majority involves quantitative assessments using the PISQ-IR or other pre-post assessments.^40–42^ Although the PISQ-IR is considered the gold standard for research on sex with prolapse, pre- and post-quantitative assessments of sexuality are limited in their ability to understand the nuances of sexuality and, as such, are unlikely to capture women’s struggles with sexuality related to POP and short- and long-term impacts of surgical repair. In our study, findings related to women’s tentative returns to sexual activity, perceived or real pressures to engage in sexual activity, and changes to body image and sexual confidence would have been missed if participants had only been assessed using the PISQ-IR. These important findings reflect the critical need for qualitative approaches, which are particularly appropriate for sex research as they allow for the exploration of nuance and complexity in experiences.^43^

### Strengths and limitations

This study had several strengths. To our knowledge, it is the first to use a longitudinal approach to qualitatively examine women’s experiences of sexuality and POP. Therefore, we were uniquely able to assess women’s experiences both before and at multiple time points after POP surgery. We were able to center their live experience of women experiencing prolapse. The study’s longitudinal design allowed a better understanding of the impacts of POP on sexuality and the return to sexual activity post-surgery. The qualitative approach allowed for discovery of new insights into the impacts of POP surgery on women’s sexuality, which can often be overlooked by self-report questionnaires. This study provides evidence to inform clinical counseling specifically regarding patient expectations of surgical repair of POP, to sexual recovery timelines after surgery, and to relative impact of prolapse on women’s sexuality pre- and post-surgery. However, this study also has several limitations. Our sample was diverse in age but not in ethnicity or socioeconomic status. It is possible that women from other ethnicities, cultural groups, or socioeconomic situations may have different experiences and expectations. The study also only focused on women who elected surgical repair of POP, and therefore, findings may be unique to this subset of women.

## Conclusion

This study highlights the complex and changing experiences of women’s sexuality prior to and after surgical repair of POP and the importance of qualitative inquiry and the inclusion of women with lived experience of POP in urogynecologic research. Overall, surgical repair of POP ultimately resulted in the resolution of certain sexual difficulties experienced pre-surgery. However, women may overestimate the role of POP in their sex lives pre-surgery, with certain challenges related to sex (e.g., attitudes, competing stressors, family demands, and relationship challenges) continuing to impact their sexuality post-surgery. Clinical counseling around the impacts of surgical repair of POP on women’s sexuality should include expectation management, including the timeline for recovery and which aspects of sexuality are reasonably expected to improve.

## Supplemental Material

sj-docx-1-whe-10.1177_17455057261437391 – Supplemental material for A longitudinal, qualitative exploration of women’s sexual recovery following surgical repair of pelvic organ prolapse Supplemental material, sj-docx-1-whe-10.1177_17455057261437391 for A longitudinal, qualitative exploration of women’s sexual recovery following surgical repair of pelvic organ prolapse by Kaylee Ramage, Erin A. Brennand and Lauren M. Walker in Women's Health
