# Supplementary material for: A longitudinal, qualitative exploration of women’s sexual recovery following surgical repair of pelvic organ prolapse
Source: Womens Health (Lond). 2026 Apr 20;22:17455057261437391. doi: 10.1177/17455057261437391 (PMC13110285; doi:10.1177/17455057261437391)
Supplement: sj-docx-1-whe-10.1177_17455057261437391 – Supplemental material for A longitudinal, qualitative exploration of women’s sexual recovery following surgical repair of pelvic organ prolapse [file sj-docx-1-whe-10.1177_17455057261437391.docx]

**Supplemental File: Abbreviated Interview Guides for Baseline, 3-month, and 6-month interviews**

**Abbreviated Baseline Interview Guide**

1. In what ways has having pelvic organ prolapse influenced or impacted your sexuality?
2. How do you perceive your pelvic organ prolapse to have impacted your body image?
3. Does the experience of having pelvic organ prolapse change the way you feel about yourself with respect to gender?
4. How has pelvic organ prolapse impacted your sense of comfort and discomfort with sex?
5. How has pelvic organ prolapse impacted your motivation to be sexual spontaneously (i.e. initiating sex with your partner or yourself)?
6. How do you perceive your pelvic organ prolapse to have impacted your receptiveness to a partner’s invitations to be sexual (i.e. accepting sex)?
7. How has pelvic organ prolapse to have impacted the context in which you are sexual?
8. How do you perceive your pelvic organ prolapse to have impacted your ability to become aroused?
9. How do you perceive your pelvic organ prolapse to have impacted what kinds of activities you are willing to engage in?
10. How do you perceive your pelvic organ prolapse to have impacted what kinds of activities bring you pleasure or enjoyment?
11. How do you perceive your pelvic organ prolapse to have impacted the rewards you stand to gain from sex?
12. How do you perceive your pelvic organ prolapse to have impacted any difficulties that might arise during or after sex?
13. Do you think your ability to orgasm is different since you developed prolapse? If so, how?
14. Are you satisfied with the amount of “friction” or “contact” that occurs with penetrative intercourse?
15. Do you think your partner’s views/thoughts on your sexuality are related at all to your prolapse?
16. What challenges come up for you sexually, specifically related to prolapse?
17. How do the challenges you just mentioned compare with other challenges that might get in the way of sex or of having satisfying sex?
18. When deciding to have surgery for prolapse, was your sexuality a part of the reasons that influenced you? If so, how?
19. With respect to your upcoming surgery, is there anything you hope your surgical procedure will do to positively affect your sexuality?
20. With respect to your upcoming surgery, do you have any worries about your surgery negatively affecting your sexuality?
21. Do you feel prepared to return to sexual activity after surgery?
22. How could your surgical team better prepare you to return to sexual activity after your upcoming procedure?
23. When you do think you will return to sexual activity after your surgery?
24. Anything else you would like to mention or talk about regarding your prolapse, treatment or sexuality?

**Abbreviated 3-month and 6-Month Follow-up Interview Guide**

1. When did you return to sexual activity after surgery? This can be by yourself or with a partner.
2. Was your first sexual experience after surgery with a partner or by yourself?
3. When the time came for your first sexual experience after surgery, did you feel prepared for the encounter?
4. How did you “know” you were ready to return to sexual activity? What were the factors that lead to the first encounter?
5. Since surgery for pelvic organ prolapse, have any aspects of your sexuality changed?
6. Has your POP surgery impacted your body image?
7. After having POP, has your sense of comfort and discomfort with sex changed in any way?
8. Did the surgery you underwent impact your motivation to be sexual spontaneously (i.e. initiating sex with your partner or yourself)?
9. How about impacting your receptiveness to a partner’s invitations to be sexual (i.e. accepting sex)?
10. Has your surgery resulted in changes to the context in which you are sexual?
11. Since your surgery for POP has your ability to become aroused changed?
12. How do you perceive having prolapse has impacted what kinds of activities you are willing to engage in?
13. How do you perceive having surgery for pelvic organ prolapse to have impacted what kinds of activities bring you pleasure or enjoyment?
14. Has surgery for prolapse impacted the rewards you stand to gain from sex?
15. How did your prolapse surgery impacted any difficulties that previously might arise during or after sex? Has it introduced any new difficulties during or after sex (such as pain or difficulties with penetration?)
16. Do you think your ability to orgasm is different since underwent your prolapse surgery? If so, how? If so, do you have any thoughts on what caused that change?
17. Now that you’ve had surgery for POP, are you satisfied with the amount of “friction” or “contact” that occurs with penetrative intercourse?
18. Since undergoing your POP surgery, do you think your partner’s views/thoughts on your sexuality have changed?
19. From your overall viewpoint, would you say surgery for prolapse affected your sexuality in a positive, negative or neutral (meaning no change) manner?
20. If a friend disclosed to you that they were experiencing POP, would you advise them to have surgery to correct it? What pieces of your surgical experience would you share to help them make that decision?
21. Now that you’ve gone through the surgical process, what advice would you give a surgical team so that we can better prepare patients to return to sexual activity after their procedures?
22. Anything else you would like to mention or talk about regarding your prolapse, treatment or sexuality?
